# Treatment of a Femur Nonunion with Microsurgical Corticoperiosteal Pedicled Flap from the Medial Femoral Condyle

**DOI:** 10.1155/2016/5125861

**Published:** 2016-03-15

**Authors:** Matteo Guzzini, Cosma Calderaro, Marco Guidi, Carolina Civitenga, Germano Ferri, Andrea Ferretti

**Affiliations:** Department of Orthopaedic and Traumatology, Sant' Andrea Hospital, Faculty of Medicine and Psychology, “Sapienza” University of Rome, Via di Grottarossa 1035, 00189 Rome, Italy

## Abstract

*Introduction*. The vascularized corticoperiosteal flap is harvested from the medial femoral condyle and it is nourished by the articular branch of the descending genicular artery and the superomedial genicular artery. This flap is usually harvested as a free flap for the reconstruction of bone defects at forearm, distal radius, carpus, hand, and recently at lower limb too.* Case Report*. A 50-year-old Caucasian man referred to our department for hypertrophic nonunion of the distal femur, refractory to the conservative treatments. The first surgical choice was the revision of the nail and the bone reconstruction with a corticoperiosteal pedicled flap from the medial femoral condyle. We considered union to have occurred 3.5 months after surgery when radiographs showed bridging of at least three of the four bony cortices and clinically the patient was able to walk with full weight bearing without any pain. At the last follow-up (25 months), the patient was completely satisfied with the procedure.* Discussion*. The corticoperiosteal flap allows a faster healing of fractures with a minimal morbidity at the donor site. We suggest that the corticoperiosteal pedicled flap graft is a reliable and effective treatment for distal femur nonunion.

## 1. Introduction

The vascularized corticoperiosteal flap was introduced by Sakai et al. in 1991 [1]. Recently, it has been reported in the treatment of bone defects up to 13 cm [2]. It is harvested from the medial femoral condyle and it is nourished by the articular branch of the descending genicular artery (DGA) and the superomedial genicular artery (SMGA) [3]. The DGA is dominant in 80% of the cases and when it is traced to its origin from the femoral artery, 8 to 12 cm long pedicle can be harvested. When the SMGA is dominant, pedicle only 3 to 4 cm in length can be harvested [4].

This flap is usually harvested as a free flap for the reconstruction of bone defects at forearm, distal radius, carpus, hand [5, 6], and recently at lower limb too [7–9].

We present a rare case of distal femur nonunions successfully treated by a corticoperiosteal harvested as pedicled flap from the medial femoral condyle.

## 2. Case Report

In March 2012, a healthy 50-year-old Caucasian male sustained a traumatic bifocal pertrochanteric and distal third shaft fracture of the left femur that was treated by another surgeon with a cephalomedullary long nail distally fixed with 2 screws. After 2 months, in May 2012, during the rehabilitation period, he suffered from the displacement of the fracture because of the loosening of the distal screws. He underwent a new operation by the same surgeon to reduce the displaced fracture and replace the distal screws. Six months after the last surgery, the fracture was still unconsolidated and he underwent pulsed electromagnetic fields and extracorporeal shockwaves therapy without success.

**Figure 1:**
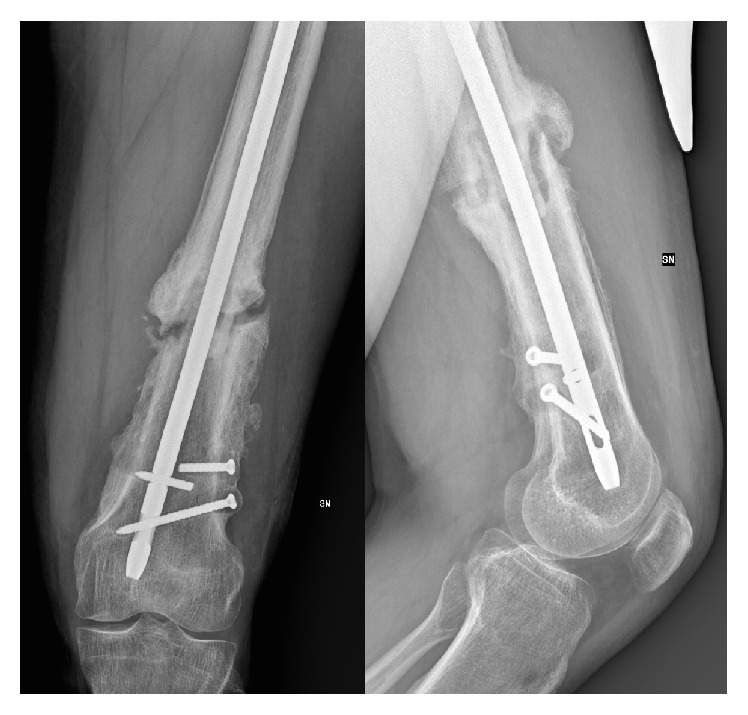
Preoperative radiographs showing an unconsolidated distal femoral shaft fracture with the rupture of one of the distal screws.

On February 2013, he referred to our department for persistent thigh pain and lameness that forced him to walk with a cane. The radiographic examination showed a hypertrophic nonunion of the distal femur (Figure 1), refractory to the conservative treatment, which has been given an indication for surgical treatment. No limitation of the knee and hip range of movement was recorded. The limb was shortened to 3 cm.

Our first surgical choice was the revision of the nail and the bone reconstruction with a corticoperiosteal pedicled flap from the medial femoral condyle.

### 2.1. Surgical Technique

Before the surgery, a CT angiography is performed to study the vascularization of the distal femur. The antibiotic preoperative profilaxis with gentamicin 160 mg (1 hour before the surgery) and cefazolin 2 g (just before the surgery) is administered.

**Figure 2:**
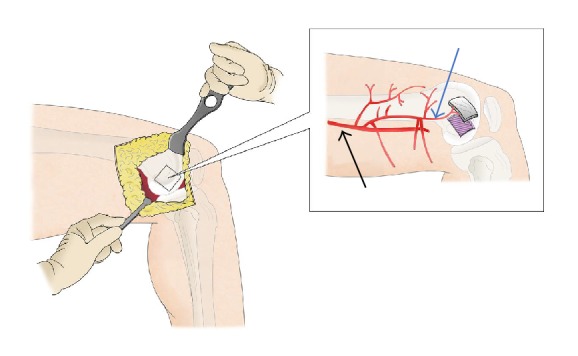
Grafting of the corticoperiosteal flap. Black arrow: femoral artery; blue arrow: descending genicular artery (drawing by G. Ferri).

**Figure 3:**
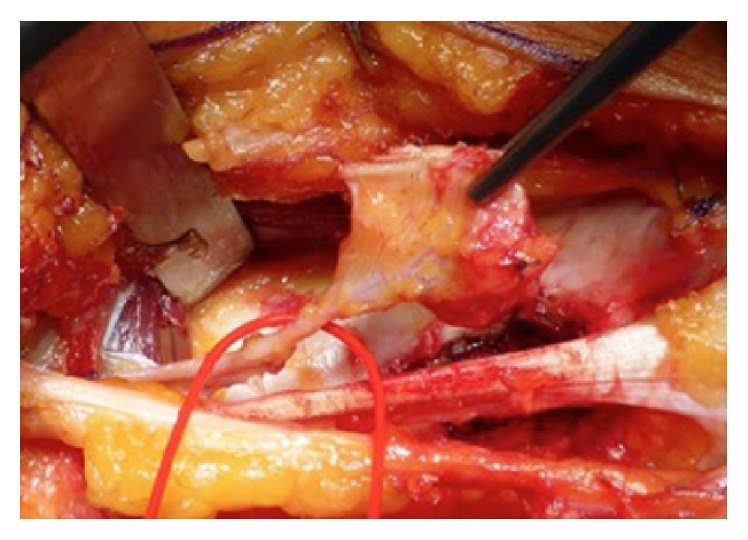
Intraoperative image of the corticoperiosteal graft.

The patient was placed in supine position with the hip and knee slightly flexed and externally rotated. A tourniquet is inflated at the proximal thigh. The removal of the intramedullary nail and screws was performed through the previous surgical access and the recipient site was prepared by debriding the bone, excising all scarred tissues and reaming of the femoral canal. A longitudinal incision was made on the medial side of the distal third of the thigh. The vastus medialis was reflected anteriorly, whereas the tendon of the adductor longus was retracted posteriorly, exposing the medial femoral condyle and its periosteal blood supply (Figure 2). The dominant vessel was identified and dissected to its source. The design of the graft was outlined on the periosteum. The periosteum was cut with an electrocautery. Large vessels were ligated with hemoclips or absorbable suture threads. Then, using an osteotome or an oscillating saw, the outer cortex was cut and lifted, hammering slightly from the periphery towards the center (Figures 2 and 3).

**Figure 4:**
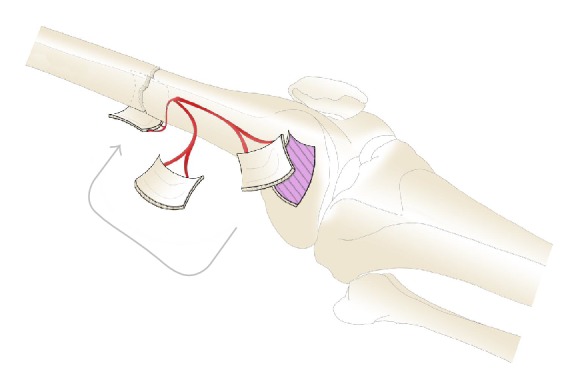
Overturning of the corticoperiosteal flap (drawing by G. Ferri).

**Figure 5:**
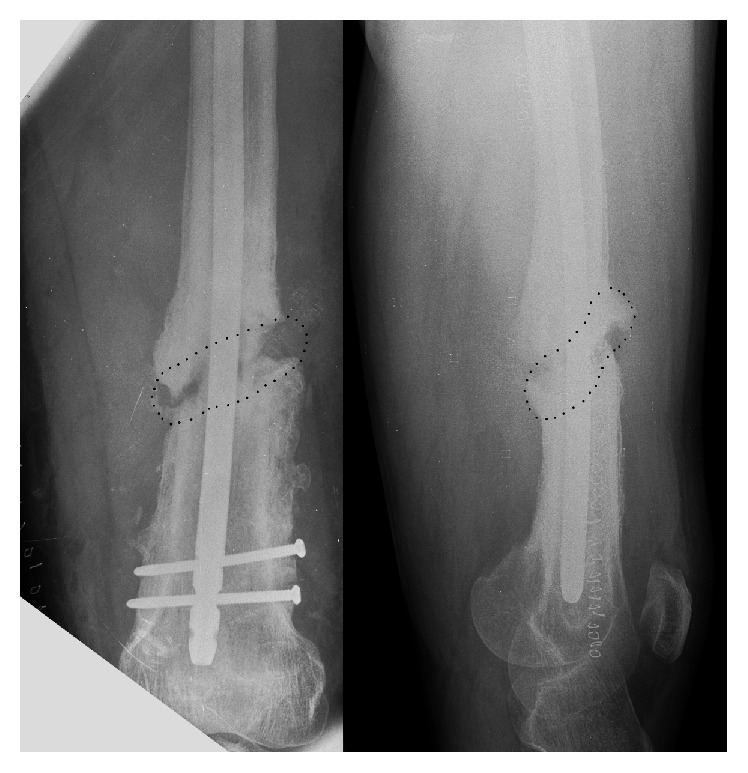
The femoral shaft fracture was stabilized with retrograde intramedullary nailing and the bone loss was restored with a corticoperiosteal pedicled flap. The X-ray shows less radiopacity of the flap (dotted line).

Simultaneously, the nail was removed from the femur bone and the recipient site was prepared by debriding the bone, excising all scarred tissues. The fracture was reduced and the osteosynthesis was performed by an anterograde intramedullary nail; the graft was overturned and wrapped around the nonunion and fixed with transbone stitches (Figure 4). A radiograph was performed at the end of the procedure (Figure 5).

### 2.2. Postoperative Period

Subcutaneous enoxaparin sodium (4000 UI) was administered as soon as the recovery of full weight bearing took place. Wound inspection was done on the first and third postoperative days. Knee and hip mobilization, quadriceps exercises, and touch-toe weight bearing were started from the third postoperative day using a frame or crutches. The patient was discharged after 7 days from the surgery and stitch removal was done on the 15th postoperative day. The patient was followed at monthly intervals up to six months, then at three monthly intervals up to one year, and then every six months up to the last follow-up. Radiological assessment was done by taking serial X-rays as needed.

Progressive to complete weight bearing was allowed on the 35th postoperative day after the appearance of callus on radiographs. The patient reached the complete weight bearing on the 60th postoperative day. We considered union to have occurred at 3.5 months of follow-up when radiologically anteroposterior and lateral radiographs showed bridging of at least three of the four bony cortices and clinically the patient was able to walk full weight bearing without any pain.

**Figure 6:**
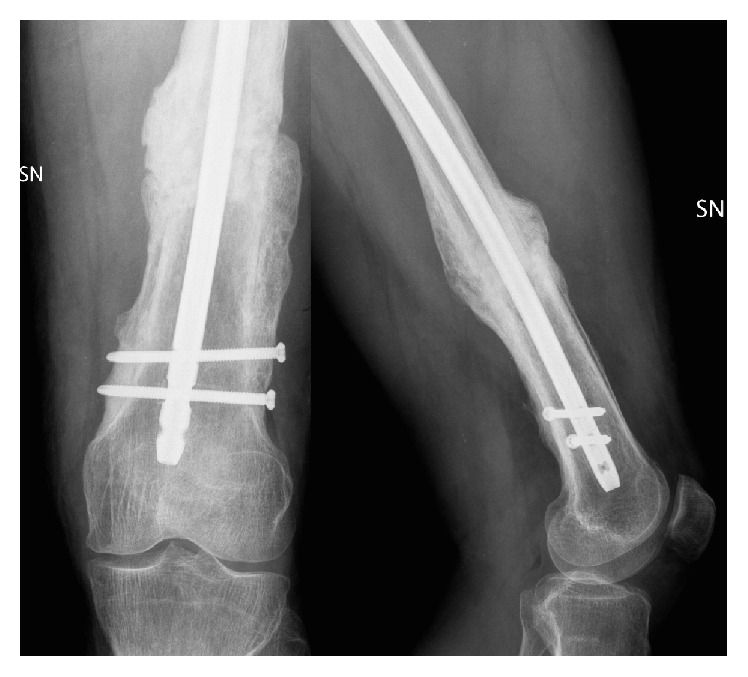
At 20 months of follow-up, the radiographic examination showed the complete healing of the femoral shaft nonunion.

At the 20 months of follow-up, there was not recorded pain or excessive intoeing/out-toeing walking and the limb was shortened to 1.5 cm compared to the opposite leg. The radiographs showed complete union of the distal femur (Figure 6). The patient was completely satisfied with the procedure.

## 3. Discussion

Fracture healing is a complex process resulting in optimal skeletal repair [10, 11]; despite this, approximately 5% of the fractures result in nonunions [12].

A lot of treatments, both conservative (low intensity ultrasound, pulsed electromagnetic fields, and extracorporeal shock waves) [13–16] and surgical (minimal screw plate fixation, external fixation, and autogenous bone like iliac crest graft and vascularized fibular graft) [17–24], have been described in order to improve the local biomechanical environment or blood supply. Among these, the corticoperiosteal flap is increasingly being used in the case of bone loss, thanks to its biological properties, flexibility, and low morbidity at the donor site [25–28].

The corticoperiosteal graft consists of periosteum and a thin (0.5–1 mm) stratum of the outer cortical bone. If required, the flap can be harvested with cancellous bone too [29]. The preservation of the osteocytes of the cambium layer (the deeper periosteal layer) accelerates graft consolidation and fracture healing, giving the corticoperiosteal flap a better osteogenic capacity than the periosteal flaps alone. Thanks to its size and flexibility, it can be introduced into an area of nonunion or wrapped around it [30].

The minimal morbidity at the donor site can be related to the surgical technique: the access to the medial femoral condyle is through a natural muscular cleavage plane, between the vastus medialis and the tendon of the adductor longus. The required size of the graft is drawn on the bare surface of the medial femoral condyle; the articular surface and the medial collateral ligament are protected when harvesting. Finally, the graft is not responsible for the ischemia of the medial femoral condyle because the centrifugal flow of blood from the medullary cavity to the cortical bone is not damaged [31–34].

Katz et al. tested the axial stability of the femur after harvesting corticocancellous flaps using a standardized composite femur model. They demonstrated that, when stressed with supraphysiologic forces, the femur retains its axial stability even after harvesting large corticocancellous flaps (up to 24 cm) from its medial aspect [35].

The clinical applications and the success of this flap have been previously described by different authors. Fuchs et al. reported 100% of bone healing in 3 patients with radiation-induced persistent atrophic nonunions of the clavicle that were ultimately healed by free vascularized corticoperiosteal bone grafts [36].

More recently, Choudry et al. described 12 cases of bone nonunions (3 of the humerus, 1 of the radius, 2 of the clavicle, 4 of the femur, and 2 of the tibia). Nine (75%) of the nonunion sites healed primarily without complication at an average period of 3.8 months, 2 nonunions healed secondarily following hardware modification; 1 flap failed due to arterial thrombosis [37].

Similar success rates have been reported by Muramatsu et al. in their treatment of 10 humeral nonunions [38].

The corticoperiosteal flap allows avoiding various complications at the donor site that can frequently occur after other bone grafts: chronic (>6 months) donor site pain (8%), dysesthesias around the incision area, iatrogenic nerve injuries (cluneal nerves and lateral femoral cutaneous nerve), superior gluteal artery injuries, iliac fractures and hernias, in case of iliac crest bone graft [39–41] chronic pain (7%), dysesthesias around the incision area, instability and limited range of motion in the ankle, sensory deficit, claw toe, and dorsiflexion of the great toe, in case of vascularized fibula graft [42].

In conclusion, the corticoperiosteal flap is harvested by a microsurgical technique. Compared with traditional grafts, thanks to its high osteogenic nature and its vascularization, the corticoperiosteal pedicled graft allows a faster healing of fractures with a minimal morbidity at the donor site. According to our experience and knowledge of the literature, we suggest that the corticoperiosteal pedicled flap graft is a reliable and effective treatment for distal femur nonunion.
